# Familial Hypercholesterolemia: From Clinical Suspicion to Novel Treatments

**DOI:** 10.31083/j.rcm2411311

**Published:** 2023-11-09

**Authors:** Saeid Mirzai, Parag Anilkumar Chevli, Rishi Rikhi, Michael D. Shapiro

**Affiliations:** ^1^Department of Internal Medicine, Cleveland Clinic, Cleveland, OH 44195, USA; ^2^Section on Hospital Medicine, Department of Internal Medicine, Wake Forest University School of Medicine, Winston-Salem, NC 27157, USA; ^3^Section on Cardiovascular Medicine, Department of Internal Medicine, Wake Forest University School of Medicine, Winston-Salem, NC 27157, USA; ^4^Center for Prevention of Cardiovascular Disease, Section on Cardiovascular Medicine, Wake Forest University School of Medicine, Winston-Salem, NC 27157, USA

**Keywords:** familial hypercholesterolemia, heterozygous, homozygous, low-density lipoprotein, atherosclerosis, cardiovascular events, diagnosis, genetic testing, cascade screening, lipid-lowering therapies

## Abstract

Familial hypercholesterolemia (FH) is the most common monogenic disorder in humans. It affects millions of people globally, increasing the risk of developing cardiovascular disease (CVD) at a younger age due to elevated levels of low-density lipoprotein cholesterol (LDL-C) from birth. While effective traditional and novel treatments are available, the most significant challenge with FH is the lack of timely diagnosis. As a result, many patients remain undertreated leading to an increased risk of CVD. To mitigate risk, initiating early and aggressive LDL-C-lowering therapies is recommended. Moreover, given its autosomal dominant inheritance patterns, it is also recommended to perform cascade lipid and/or genetic testing of all first-degree relatives. This review highlights the importance of early FH diagnosis and available treatment options. Greater awareness and improved screening efforts can help diagnose and treat more individuals, ultimately reducing the CVD risk associated with FH.

## 1. Introduction

**Fig. 1.**
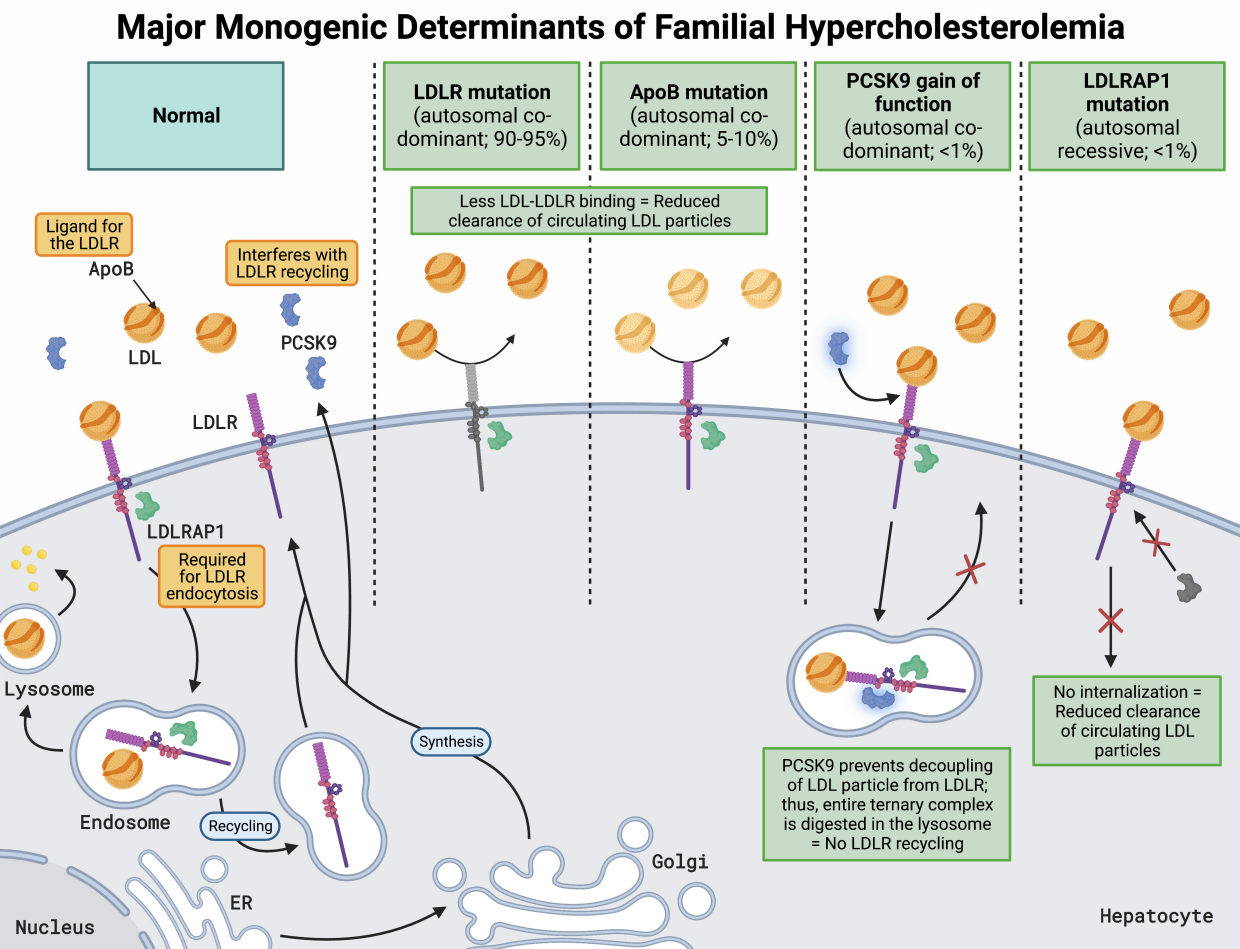
**Major monogenic determinants of familial hypercholesterolemia [3, 4, 5, 6, 7, 8]**. LDLRs are located on the hepatocyte surface and bind LDL via apoB, resulting in receptor-mediated endocytosis facilitated by LDLRAP1. The LDL-LDLR complex is then transported to an early endosome which acidifies, resulting in a conformational change of the LDLR and subsequent offloading of the LDL particle. The endosome carrying the now free LDL particle fuses with a lysosome, where its lipid cargo is repurposed in a myriad of ways depending on cellular needs. At the same time, the LDLR is recycled back to the hepatocyte surface for further rounds of LDL particle clearance. Another key regulator of LDLR recycling and cholesterol homeostasis is PCSK9, a low-abundance plasma protein that is secreted predominantly by the liver and can bind to the LDLR. With PCSK9 bound to LDLR, the same process of clathrin-mediated receptor endocytosis occurs; however, PCSK9 prevents the conformational change in LDLR from occurring. Without the structural change in LDLR, the LDL particle is not offloaded, and the entire LDL-PCSK9-LDLR complex fuses with a lysosome for destruction, preventing LDLR recycling. Thus, loss of function of *LDLR*, *apoB*, and *LDLRAP1*, or gain in function of *PCSK9*, results in decreased LDL plasma clearance, leading to FH. Abbreviations: LDLR, low-density lipoprotein receptor; LDL, low-density lipoprotein; apoB, apolipoprotein B; LDLRAP1, LDLR adaptor protein-1; PCSK9, proprotein convertase subtilisin/kexin type 9; ER, endoplasmic reticulum; Golgi, Golgi apparatus.

Familial hypercholesterolemia (FH) is the most common monogenic disorder in humans, affecting 25–30 million individuals worldwide [1]. It is an inherited autosomal dominant disorder that impacts low-density lipoprotein (LDL) particle clearance, resulting in elevated LDL cholesterol (LDL-C) [2]. Genetic mutations in the LDL receptor (*LDLR*) account for the majority of FH cases, while mutations in apolipoprotein B (*apoB*) and proprotein convertase subtilisin/kexin type 9 (*PCSK9*) account for a minority [3]. Additionally, mutations in the LDLR adaptor protein-1 result in a rare form of autosomal recessive FH (Fig. 1, Ref. [3, 4, 5, 6, 7, 8]) [3].

FH is either categorized as heterozygous, one allele impacted, or as the much rarer homozygous form, with two pathological allelic variants. Homozygous FH (HoFH) typically refers to the same mutation affecting both alleles of one of the FH-causing genes, but patients can also be compound heterozygous with a different mutation at each allele of one of the FH-causing genes. Regardless of the genetics, these gene mutations exhibit additive effects with both types resulting in severe hypercholesterolemia [9]. In the general population, heterozygous FH (HeFH) impacts approximately 1:313 individuals, while HoFH, considered an orphan disease, impacts approximately 1:400,000 individuals [2, 10, 11]. LDL-C values are highly variable and range from double to up to six times greater in those with HeFH and HoFH, respectively, compared to the general population [2]. Furthermore, and perhaps more importantly, individuals with FH have more prolonged arterial exposure to LDL from birth [4]. Therefore, patients with FH exhibit atherosclerosis earlier in life and have a greater prevalence of atherosclerotic cardiovascular disease (ASCVD) [4]. Natural history studies in individuals with HeFH, before statins, have estimated the risk of major cardiovascular (CV) events (such as fatal or non-fatal coronary events) to be 50% in men by 50 years and 30% in women by 60 years of age, if untreated [5, 12]. In those with HoFH, events can occur as early as childhood [13].

It is important to note significant global disparities in the diagnosis and treatment of FH, where the average time before a clinical ASCVD event in those with HoFH is approximately ten years earlier in low-income countries compared to high-income countries [14]. Additionally, FH prevalence varies by ethnicity, with the greatest prevalence in Black individuals and the lowest prevalence in Asian individuals (blacks 1:192, whites 1:323, Asians 1:400) [15, 16]. Furthermore, important differences in FH outcomes exist based on sex, with women being less likely to receive high-intensity statin therapy and attain guideline LDL-C goals [17].

This review highlights the importance of diagnosis, genetic screening, and early initiation of lipid-lowering treatments for individuals with FH. Furthermore, we discuss the current literature supporting traditional and novel therapies available for managing these patients.

## 2. Clinical Presentation and Complications

Patients with FH have increased LDL-C levels from birth, leading to a higher prevalence and earlier onset of ASCVD with associated signs or symptoms, including angina, myocardial infarction, stroke, transient ischemic attack, or claudication [13, 18, 19]. For example, coronary artery disease (CAD) prevalence was 47% in adult men and 30% in adult women with FH included in the Cascade Screening for Awareness and Detection (CASCADE) Registry, which is 5 to 7 times greater than the age-matched general United States population [18]. The Spanish Familial Hypercholesterolemia Cohort Study (SAFEHEART) Registry data also demonstrated a threefold higher prevalence of ASCVD in these patients compared with their unaffected relatives [19]. Patients with HoFH experience an even greater accelerated process of atherosclerosis. Based on the amount of activity in skin fibroblasts, HoFH patients are classified as receptor-null (<2% of normal LDLR activity) or receptor-defective (2% to 25% of normal LDLR activity) [20]. LDLR-null HoFH patients rarely live beyond their second decade without treatment. LDLR-defective patients have a better prognosis; however, nearly all of these patients will develop clinically significant ASCVD by 30 years of age or earlier if untreated [13, 20].

The objective of the physical examination is to discover excessive cholesterol deposits in the tendons and eyes. The presence of tendon xanthomas, which are depositions of cholesterol in connective tissues, is pathognomonic of FH [21]. The Achilles tendon is the most common location for tendon xanthomas, followed by the extensor tendons of the hand and the patellar tendons. Tendon xanthomas in HoFH are typically noticeable by age 10 [22]. However, in HeFH, these xanthomas may not become evident until early adulthood. In fact, the incidence of the physical stigmata of FH has decreased in recent years. The reason for this observation is unknown, though it may be due to the more ubiquitous use of statins for hypercholesterolemia in general [22, 23]. Other manifestations include corneal arcus (more specific for FH when identified in individuals <35 years old), a white or gray opaque ring along the corneal border, and xanthelasma - yellowish papules and plaques caused by a localized accumulation of lipid deposits commonly seen on the eyelids. However, xanthelasma is not diagnostic because it can be found in individuals without FH, but it should raise suspicion in the appropriate clinical context [24]. In addition, patients with HoFH may present with aortic stenosis caused by various factors, including cholesterol and calcium deposits, aortic root inflammation, and fibrosis of the aortic valve cusps [25].

Before making a clinical diagnosis of FH, it is essential to exclude secondary causes of severe hypercholesterolemia, such as hypothyroidism, nephrotic syndrome, liver disease, and diabetes. It is also important to note that other genetic disorders of lipid metabolism may overlap with the FH phenotype, including polygenic hypercholesterolemia, familial combined hyperlipidemia, familial dysbetalipoproteinemia, and sitosterolemia. Tendon xanthomas are also observed in sitosterolemia, a rare autosomal-recessive disorder that resembles FH but responds dramatically to dietary modifications and/or ezetimibe [26].

## 3. Diagnosis and Screening

A clinical diagnosis of FH is based on a combination of physical findings, personal or family history of hypercholesterolemia, early-onset ASCVD, and circulating LDL-C concentration [5]. Multiple sets of criteria consisting of clinical, biochemical, and genetic characteristics have been developed and used widely to diagnose FH (Table 1, Ref. [27]; Table 2, Ref. [28]; Table 3, Ref. [29]) [6]. There are currently four accepted resources available to diagnose FH: (1) The Dutch Lipid Clinic Network criteria; (2) The Simon Broome criteria; (3) The United States Make Early Diagnosis to Prevent Early Death (MEDPED) criteria; and (4) The American Heart Association (AHA) diagnostic criteria. The Dutch Lipid Clinic Network criteria is the most widely used of these strategies. It provides a score based on family and personal history of premature ASCVD, physical examination findings, LDL-C level, and genetic testing (Table 1) [27]. Utilized mainly in the United Kingdom, the Simon Broome criteria define patients as having either definite or probable FH (Table 2) [28]. In contrast to the Dutch Lipid Clinic Network criteria, a positive genetic test result is sufficient to establish a definite diagnosis of FH according to the Simon Broome criteria. The United States MEDPED primarily utilizes age and relative-specific parameters for total cholesterol cut-off points (Table 3) [29]. If there is no history of FH in the family, the diagnostic threshold is based on the level in the general population. The MEDPED criteria are simple to employ but do not account for clinical examination or genetic testing. The Dutch Lipid Clinic Network and Simon Broome criteria depend on the existence of physical signs of FH, which reduces their diagnostic effectiveness. These algorithms have been calibrated for higher specificity/lower sensitivity to facilitate cascade lipid and/or genetic testing. Due to modest sensitivity, these algorithms are suboptimal for index case identification. Moreover, these schemata are hindered by complex criteria that are difficult to apply in a clinical setting. The AHA criteria were developed to address these practical challenges, improve the identification of index cases, and simplify the process by relying on LDL-C levels and family history [7]. The National Lipid Association expert panel provided recommendations for screening and diagnosing FH based on LDL-C and non-high-density lipoprotein cholesterol (non-HDL-C) [30]; however, it was not intended to substitute any of the validated criteria.

**Table 1.** **Dutch Lipid Network Criteria for FH [27]**.

Criteria		Score
Family history		
	First-degree relative with ${\mathrm{premature}}^{*}$ cardiovascular disease OR	1
	First-degree relative with LDL-C >95th percentile	
	First-degree relative with tendon xanthomas or arcus cornealis, OR	2
	Child under age 18 with LDL-C >95th percentile	
Clinical history		
	${\mathrm{Premature}}^{*}$ coronary artery disease	2
	${\mathrm{Premature}}^{*}$ cerebral or peripheral vascular disease	1
Physical examination		
	Tendon xanthomas	6
	Arcus cornealis before age 45	4
LDL-C levels		
	≥330 mg/dL	8
	250–329 mg/dL	5
	190–249 mg/dL	3
	155–189 mg/dL	1
DNA analysis		
	Functional mutation in the *LDLR*, *apoB*, or *PCSK9* gene	8
Diagnosis based on the total score		Total
	Definite FH	>8
	Probable FH	6–8
	Possible FH	3–5
	Unlikely FH	<3

*apoB*, apolipoprotein B; FH, familial hypercholesterolemia; LDL-C, low-density lipoprotein cholesterol; *LDLR*, low-density lipoprotein receptor; *PCSK9*, proprotein convertase subtilisin/kexin type 9. ^*^Premature = <55 years in men; <60 years in women.

**Table 2.** **Simon Broome Diagnosis Criteria for FH [28]**.

Diagnosis	Criteria	
Definite FH		Total Cholesterol >290 mg/dL or LDL-C >190 mg/dL in adult
		Total Cholesterol >260 mg/dL or LDL-C >155 mg/dL in children
	PLUS	
		Tendon xanthomas in a patient or a first-degree relative (parent, sibling, child) or in a second-degree relative (grandparent, uncle, aunt)
	OR	
		DNA-based evidence of a functional *LDLR*, *PCSK9*, or *apoB* mutation
Probable FH		Total Cholesterol >290 mg/dL or LDL-C >190 mg/dL in adult
		Total Cholesterol >260 mg/dL or LDL-C >155 mg/dL in children
	PLUS	
		Family History of myocardial infarction before 50 years of age in a second-degree relative or below age 60 in a first-degree relative
	OR	
		Family history of Total Cholesterol >290 mg/dL in a first- or second-degree relative

*apoB*, apolipoprotein B; FH, familial hypercholesterolemia; LDL-C, low-density lipoprotein cholesterol; *LDLR*, low-density lipoprotein receptor; *PCSK9*, proprotein convertase subtilisin/kexin type 9.

**Table 3.** **United States MEDPED Criteria for FH [29]**.

	Total cholesterol cut-off points in mg/dL			
Age (years)	First-degree relative with FH	Second-degree relative with FH	Third-degree relative with FH	General Population
<20	220	230	240	270
20–29	240	250	260	290
30–39	270	280	290	340
≥40	290	300	310	360

FH, familial hypercholesterolemia; MEDPED, Make Early Diagnosis to Prevent Early Death. FH is diagnosed if total cholesterol exceeds these cut-off points.

These algorithms do not apply to patients with HoFH except the AHA diagnostic schema, which incorporates distinct diagnostic criteria for HoFH. The LDL-C >500 mg/dL if untreated (treated LDL-C >300 mg/dL) and evidence of xanthoma in the first decade of life, or increased LDL-C levels consistent with HeFH in both parents, are diagnostic criteria for HoFH [13].

### 3.1 Genetic Testing for FH

Genetic testing for FH aims to provide an underlying molecular diagnosis and offer valuable prognostic information. It is currently advocated globally, including by the American College of Cardiology, International Societies of Atherosclerosis, and European Societies of Atherosclerosis [12]. Although genetic testing is performed extensively at the population level in European countries, the CASCADE Registry results demonstrated that genetic testing for FH was underutilized in the United States, with only 4% of participants with a clinical diagnosis of FH reporting genetic testing [31]. Mutations in the *LDLR*are by far the most frequent cause of FH [32], but genes encoding apoB and PCSK9 are also associated with FH [7]. Mutations in one of these three genes account for 60–80% of cases of FH [3]. In those with genetically confirmed FH, the vast majority (90–95%) of identified mutations are found in the *LDLR* gene, 5–10% in the *apoB* gene, and approximately 1% in the *PCSK9* gene (Fig. 1) [6, 7, 8].

The utility of the established clinical criteria for diagnosing FH has been challenged previously due to the phenotypic heterogeneity of these patients. For example, a study of the Dutch Lipid Clinic network database identified 2400 patients as having FH using established clinical diagnostic criteria, but showed significantly different clinical and laboratory profiles between those with versus without a known *LDLR* mutation [33]. In addition, the limitations of employing specific LDL-C cutpoints to identify individuals with pathogenic FH mutations have been highlighted. A whole-exome sequencing analysis from a large cohort, including participants from seven case-control studies (CAD case subjects and CAD-free control subjects) and five prospective cohort studies, demonstrated that 45% of those with LDL-C levels ≥190 mg/dL and 27% with LDL-C level <130 mg/dL had a pathogenic variant [34]. Thus, one purpose of FH genetic testing is to identify individuals who may not have been diagnosed with FH based on their lipid levels, clinical and physical characteristics, and/or family history [12].

Genetic testing is also valuable for providing prognostic information. In a study including 26,025 participants, compared with a reference group with LDL-C <130 mg/dL and no mutation, there was a 6-fold higher risk for CAD in participants with LDL-C ≥190 mg/dL and no FH mutation and a 22-fold higher risk in those with both LDL-C ≥190 mg/dL and an FH mutation [34]. Similarly, a study including 409 FH patients participating in the Simon Broome British Heart Foundation study showed that compared with those with no mutation, the participants with *LDLR* mutation had 70% higher odds of CAD, and those with *PCSK9* mutation had 20-fold higher odds of CAD [35]. Another study examined the effect of monogenic and polygenic causes of hypercholesterolemia on the incidence of early ASCVD in patients with clinically diagnosed FH and demonstrated that a monogenic cause of FH was associated with a significantly increased risk of CV disease (CVD) (hazard ratio (HR): 1.96; 95% confidence interval (CI): 1.24–3.00; *p* = 0.004), while patients with polygenic FH did not differ significantly from those with no identified genetic cause of FH [36]. These studies suggest that those with FH-causing mutations may have a higher average lifetime LDL-C exposure than those without a mutation, which might contribute to higher ASCVD risk among mutation carriers. FH genetic testing should be followed by pre and post-test genetic counseling to ensure that patients are informed of the advantages, disadvantages, and familial ramifications of genetic testing [12].

### 3.2 Cascade Testing for FH

Considering the autosomal dominant pattern of FH, it is essential to identify other family members with FH. Cascade testing is a mandatory part of the approach in which LDL-C measurement, genetic testing, or both are performed on all first-degree relatives of FH patients [7]. It has been demonstrated that cascade testing leads to earlier FH detection and is a cost-effective strategy for reducing CAD, myocardial infarction, and death [37, 38]. An analysis of the SAFEHEART registry revealed that cascade testing prevented 847 coronary events and 203 deaths in 9000 FH patients over a 10-year follow-up period, resulting in an additional 767 quality-adjusted life years [38]. In addition, the data from the United States population using a simulation model demonstrated that cascade genetic testing for FH was cost-effective if started before age 40 in first-degree relatives and before age 15 in second-degree relatives [39]. Not only is cascade testing cost-effective, but it is also crucial because FH is eminently treatable [40]. When detected and treated at a young enough age, the risk of ASCVD can be drastically reduced, possibly to average levels. Although the Centers for Disease Control and Prevention have given cascade testing of relatives of patients with FH the Tier 1 classification, there are currently no systematic cascade testing programs in the United States [41]. In addition to reluctance on the part of family members to accept genetic testing, the lack of trained healthcare professionals to perform the necessary pedigree construction and the logistics of contacting relatives to obtain informed consent for genetic testing and a blood sample, are the primary obstacles to the cascade testing [42].

### 3.3 Risk Stratification for FH

Although patients with FH are regarded to be at high ASCVD risk, the evidence indicates that their CV prognosis is quite heterogeneous. Despite substantial research into the factors influencing ASCVD risk in the FH population, precise risk prediction remains unclear [43]. There are several risk calculators explicitly designed for the FH population [44, 45, 46]. The Montreal-FH-SCORE incorporates five clinical variables, including age, sex, smoking, hypertension, and HDL-C, and has been validated in retrospective cohorts [47]. The SAFEHEART risk equation, which constitutes traditional risk factors such as age, sex, smoking, hypertension, body mass index, a history of ASCVD, and levels of LDL-C and lipoprotein (a) (Lp(a)), has also been externally validated but included both primary and secondary CV prevention populations [48]. Recently, a new score called the FH-Risk-Score was developed to predict incident ASCVD events in a large multinational prospective cohort of patients with FH without prior history of ASCVD that incorporates seven clinical variables, including age, sex, hypertension, smoking, LDL-C, HDL-C, and Lp(a) [46]. It was found to be a better predictor of future ASCVD events than the SAFEHEART risk equation for FH patients with no prior history of ASCVD [46]. To improve CV risk stratification in FH, the detection of subclinical atherosclerosis utilizing coronary artery calcium, carotid intima-media thickness, and coronary computed tomographic angiography has also been proposed [49, 50].

## 4. Treatment Goal

Lack of early detection remains the biggest challenge in FH care [8]. Once diagnosed, the primary goal of therapy in FH is aggressive LDL-C lowering, which is shown to decrease the atheroma burden [51] and prevent CV events [52, 53]. Lifestyle modifications should be encouraged, but whilst mandatory, they are typically insufficient to adequately lower LDL-C levels. Therefore, lifestyle changes should be started in tandem with lipid-lowering therapies (LLT) (Table 4, Ref. [54]). It is crucial that LLT be initiated as soon as possible after diagnosis; or starting at 8–10 years of age in children (earlier for extreme LDL-C elevations or other major risk factors) [55].

**Table 4.** **Lipid-lowering treatments for familial hypercholesterolemia [54]**.

Medication	Mechanism of Action	FDA Approval Date	Route of Administration	LDL-C Lowering in HeFH (%)	LDL-C Lowering in HoFH (%)	Common Side Effects
Oral Therapies						
Statins	HMG-CoA reductase inhibitor	1987	Oral	50–60	10–25	Nasopharyngitis, myalgia, arthralgia, diarrhea, pain in extremities, UTI
Ezetimibe	NPC1L1 inhibitor	2002	Oral	15–25	<10	Nasopharyngitis, myalgia, URI, diarrhea, arthralgia, sinusitis, pain in extremities
Bempedoic acid	ACL inhibitor	2020	Oral	15–20	-	URI, muscle spasms, hyperuricemia, back pain, abdominal pain, bronchitis, pain in extremities, anemia, elevated transaminases
Lomitapide	MTP inhibitor	2012	Oral	-	20–50	Diarrhea, nausea, vomiting, dyspepsia, abdominal pain
Injectable Therapies						
Evolocumab and alirocumab	Monoclonal antibody against PCSK9	2015	Subcutaneous injection	50–60	20–30	Nasopharyngitis, URI, influenza, back pain, injection site reactions
Inclisiran	siRNA targeting PCSK9	2020	Subcutaneous injection	40–60	-	Injection site reaction, arthralgia, UTI, diarrhea, bronchitis, pain in extremities, dyspnea
Evinacumab	ANGPTL3 inhibitor	2021	Intravenous infusion	50	50	Nasopharyngitis, influenza-like illness, dizziness, rhinorrhea, nausea

**Abbreviations**: HMG-CoA, 3-hydroxy-3-methylglutaryl coenzyme A; UTI, urinary tract infection; NPC1L1, Niemann-Pick C1-Like 1; URI, upper respiratory tract infection; PCSK9, proprotein convertase subtilisin/kexin type 9; siRNA, small interfering RNA; ACL, adenosine triphosphate-citrate lyase; MTP, microsomal triglyceride transfer protein; ANGPTL3, angiopoietin-like 3; FDA, Food and Drug Administration; LDL-C, low-density lipoprotein cholesterol; HeFH, heterozygous familial hypercholesterolemia; HoFH, homozygous familial hypercholesterolemia.

Treatment of FH (HeFH or HoFH) starts with high-intensity statin therapy, in most cases combined with ezetimibe, a PCSK9 inhibitor, or both [55]. Several LDL-C treatment goals have been proposed without a consensus. Most guidelines recommend a ≥50% reduction in LDL-C in adults with HeFH, with goals of <100 mg/dL in those without and <70 mg/dL in those with ASCVD or another major risk factor (the goal for adults with HoFH is as low as tolerated). The European Society of Cardiology and the European Atherosclerosis Society, in their 2019 guidelines, recommended ≥50% reduction of LDL-C from baseline with specific LDL-C goals of <135 mg/dL in children >10 years of age, <70 mg/dL in adults, and <55 mg/dL in adults with prior history of ASCVD or another major risk factor (such as diabetes or chronic kidney disease) [55]. Referral to a lipid specialist is recommended for patients with HeFH with inadequate lipid control and all patients with HoFH.

## 5. Oral Therapies

### 5.1 Statins

The treatment of FH begins with the maximally tolerated dose of a high-intensity statin, a competitive inhibitor of 3-hydroxy-3-methylglutaryl coenzyme A (HMG-CoA) reductase [56]. These drugs interfere with the rate-limiting step in cholesterol biosynthesis by antagonizing HMG-CoA reductase, thus decreasing intrahepatic cholesterol and increasing LDLR expression, which lowers circulating LDL-C through increased hepatic uptake. Statin monotherapy has been shown to reduce LDL-C by 50–60% in HeFH [57] and 10–25% in HoFH [13], along with a reduction in CV events and mortality [52, 53]. Despite pharmacodynamic concerns for statin efficacy in receptor-negative HoFH, these patients do respond to statin therapy, although to a lesser extent (23.5% LDL-C reduction in those with residual LDLR activity versus 14% in those with two null LDLR mutations) [58]. Statins have also proven safe and effective in children [59], with current recommendations to start low and gradually increase the dose to reach the LDL-C goal [55]. However, adequate lipid lowering is rarely achieved with statin monotherapy in adults with FH, thus requiring a combination with drugs utilizing alternate mechanisms of action.

### 5.2 Ezetimibe

Ezetimibe is the most commonly prescribed LDL-C-lowering drug after statins. It acts by inhibiting the Niemann-Pick C1-Like 1 transporter, which prevents intestinal uptake of dietary and biliary cholesterol and decreases cholesterol delivery to the liver, thus upregulating LDLR and increasing hepatic LDL-C uptake. Ezetimibe is affordable, well tolerated, and has outcomes benefits, as seen in the large Improved Reduction of Outcomes: Vytorin Efficacy International Trial (IMPROVE-IT) [60]. This trial found that, over a median six-year follow-up, patients with recent acute coronary syndrome (ACS) randomized to simvastatin plus ezetimibe versus simvastatin plus placebo had lower rates of the composite outcome of CV death, myocardial infarction, hospital admission for unstable angina, coronary revascularization 30 or more days after randomization, or stroke (HR: 0.94, 95% CI: 0.89–0.99), with similar adverse events [60]. An additional 20% LDL-C reduction can be expected by adding ezetimibe to statin therapy in FH patients [61, 62, 63].

### 5.3 Bempedoic Acid

Bempedoic acid is an inhibitor of adenosine triphosphate citrate lyase, an enzyme upstream of HMG-CoA reductase [64]. It lowers LDL-C through a mechanism similar to statins by interfering with intrahepatic cholesterol biosynthesis, resulting in the upregulation of LDLR on the hepatocyte surface. However, given that it is a prodrug activated only in the liver, the incidence of muscle-related adverse events is the same as seen in the placebo arm of several trials [65]. The Cholesterol Lowering via Bempedoic Acid, an ACL-Inhibiting Regimen (CLEAR) Harmony and CLEAR Wisdom phase 3 trials compared this drug to placebo in patients with ASCVD or HeFH on maximally tolerated statin therapy and found significant placebo-corrected LDL-C reductions of 18.1% and 17.4%, respectively, at 12 weeks [66, 67]. Adverse events were similar between the two groups, but higher rates of uric acid elevation were seen in the CLEAR Wisdom treatment group. Patients with HeFH made up a minority of the patients, with a pooled analysis showing greater placebo-controlled reductions of 22.3% in patients with HeFH (n = 112) as compared to 18.3% in patients without HeFH (n = 2897) [68]. This drug, along with a combination single-tablet bempedoic acid-ezetimibe, have been approved by the United States Food and Drug Administration (FDA) as an adjunct to diet and maximally tolerated statin therapy in adults with HeFH or established ASCVD requiring additional LDL-C lowering. The CLEAR Outcomes trial recently compared bempedoic acid to placebo among patients with either established or at high risk for ASCVD and intolerant to statin therapy, demonstrating greater LDL-C reduction (21.1% reductions at six months in favor of bempedoic acid) and fewer major adverse CV events (HR: 0.87, 95% CI: 0.79–0.96) with bempedoic acid. This medication has not been assessed in patients with HoFH.

### 5.4 Lomitapide

Lomitapide is an inhibitor of the microsomal triglyceride transfer protein, which plays an important role in lipoprotein synthesis in enterocytes and hepatocytes. Through the prevention of lipid transfer, lomitapide leads to reduced apoB-containing lipoprotein production in the small intestine (chylomicrons) and liver (VLDL-C) [69]. The LDLR-independent mechanism of lomitapide confers an advantage in patients with HoFH, hence its FDA approval for lipid-lowering in this population as an adjunct to a low-fat diet and other LLTs, including LDL apheresis. The efficacy and safety of lomitapide were studied in an open-label, phase 3, non-randomized, dose-escalating study of 29 patients above the age of 18 with HoFH [70]. The study demonstrated a potent LDL-C reduction of 50% at 26 weeks, 44% at 52 weeks, and 38% at 78 weeks. The adverse events were dose-dependent and included gastrointestinal symptoms, increased transaminase levels, and hepatic fat accumulation, with the drug carrying a boxed warning for risk of hepatotoxicity given the latter two events. The medication is both a substrate and inhibitor of cytochrome P450 3A4 with the potential for drug-drug interactions with other inhibitors of the enzyme, including statins and warfarin [69]. Finally, lomitapide is contraindicated in pregnancy since it may cause fetal harm. Given the above reasons, lomitapide prescribing in the United States is limited to physicians registered in a Risk Evaluation and Mitigation Strategy program. Lomitapide’s long-term efficacy and safety remain under investigation in the Lomitapide Observational Worldwide Evaluation Registry (LOWER) [71].

## 6. Injectable Therapies

### 6.1 Proprotein Convertase Subtilisin/Kexin Type 9 Inhibitors

PCSK9 is primarily produced in the liver and is secreted as a low-abundance plasma protein. It binds LDLR on the surface of hepatocytes, leading to its lysosomal degradation and decreased quantity of LDLR on the hepatic surface. Treatments targeting PCSK9 include monoclonal antibodies (evolocumab and alirocumab) that bind PCSK9, promoting its degradation, or a small interfering ribonucleic acid molecule (inclisiran) that inhibits translation of *PCSK9* mRNA, blocking its synthesis [72]. Both approaches to PCSK9 inhibition lead to greater LDLR recycling, thus increasing hepatic LDL-C uptake [20]. Generally, a further decrease in LDL-C of 50–60% can be expected with the addition of PCSK9-inhibiting monoclonal antibodies in patients with HeFH on conventional LLT [73, 74, 75, 76, 77, 78]. In patients significantly above LDL-C goal on statin monotherapy, PCSK9 inhibition may be initiated directly in lieu of trialing ezetimibe. The PCSK9 monoclonal antibodies are well tolerated across trials, with the most common adverse events being injection site reactions, mild cold or flu-like symptoms, nasopharyngitis, and myalgias [79].

#### 6.1.1 Monoclonal Antibodies

There are two large dedicated cardiovascular outcomes trials, one for each anti-PCSK9 monoclonal. The Further Cardiovascular Outcomes Research With PCSK9 Inhibition in Subjects With Elevated Risk (FOURIER) trial enrolled 27,564 patients with stable vascular disease on optimized statin therapy who had an LDL-C greater than 70 mg/dL and randomized them to either evolocumab or placebo [79]. The study demonstrated that evolocumab, compared to placebo, was associated with 59% LDL-C reduction and 15% relative risk reduction in the primary composite outcome [79]. Although this study found no overall mortality benefit, after a median of 8.4 total years (compared to 2.2 years in the initial study), the follow-up FOURIER-Open Label Extension study found patients assigned to evolocumab to have a 23% lower risk of CV death [80]. The outcomes efficacy of alirocumab was assessed in the Evaluation of Cardiovascular Outcomes After an Acute Coronary Syndrome During Treatment With Alirocumab (ODYSSEY OUTCOMES) trial, which showed a 15% reduction in all-cause mortality compared with placebo among 18,924 patients with recent ACS on background high-intensity statin therapy [81].

There have been no such CV outcomes trials for PCSK9 inhibition in FH; however, several trials have shown benefits for surrogate outcomes, such as LDL-C lowering. The PCSK9 Inhibition with Evolocumab in Heterozygous Familial Hypercholesterolaemia (RUTHERFORD-2) trial found a 60% reduction in LDL-C in participants with HeFH (n = 331) on stable LLT randomized to evolocumab compared to placebo [73]. Similarly, the Inhibition of PCSK9 with Evolocumab in Homozygous Familial Hypercholesterolaemia (TESLA Part B) trial demonstrated a 30.9% LDL-C reduction in participants HoFH patients (n = 50) on stable lipid-regulating therapy randomized to evolocumab compared to placebo. As described earlier, residual LDLR activity impacted the response to PCSK9 inhibition with blunted LDL-C reduction compared to placebo in those with one LDLR defective and one LDLR null mutation (–24.5% comparing three patients in placebo with six in the evolocumab group) versus those with two LDLR defective mutations (–46.9% comparing five patients in placebo with eight patients in the evolocumab group) [74].

The ODYSSEY FH I and FH II trials assessed the LDL-C lowering efficacy of alirocumab in HeFH patients (n = 735) on maximally tolerated LLT, and found greater LDL-C reduction in those randomized to alirocumab compared to placebo at 24 weeks (57.9% in FH I and 51.4% in FH II) [75]. Alirocumab was also assessed in HeFH with high CV risk (ODYSSEY LONG TERM), HeFH with LDL-C ≥160 mg/dL (ODYSSEY HIGH FH), and HeFH undergoing regular lipoprotein apheresis (ODYSSEY ESCAPE), with all trials meeting their primary endpoints of LDL-C reduction, LDL-C reduction, and reduced rate of apheresis treatments, respectively [76, 77, 78]. Finally, alirocumab was studied in HoFH patients in the ODYSSEY HoFH trial with a 35.6% placebo-corrected LDL-C reduction at 12 weeks [82].

#### 6.1.2 Small Interfering Ribonucleic Acid-Based GeneSilencing

Inclisiran is a small interfering ribonucleic acid that targets the liver to prevent *PCSK9* mRNA translation. It has a long half-life, allowing twice-yearly dosing for presumed better medication adherence. In phase 3 clinical trials, inclisiran led to approximately 50% reductions in LDL-C in individuals with HeFH (ORION-9) or ASCVD/ASCVD equivalents (ORION-10 and ORION-11) already on maximally tolerated LLT [83, 84]. The ORION-9 trial demonstrated a 47.9% placebo-corrected LDL-C reduction at 510 days in patients with HeFH and LDL-C levels ≥100 mg/dL, despite a maximal statin dose with or without ezetimibe (n = 482) [83]. The drug was well-tolerated in the trial, with injection-site reactions being the most common adverse events (17% treatment versus 1.7% placebo); 90.2% of these reactions were graded as mild. Future studies will be evaluating inclisiran in adolescent HeFH (ORION-16, NCT04652726) and HoFH (ORION-5, NCT03851705) patients.

### 6.2 Evinacumab

Evinacumab is a fully human monoclonal antibody targeted against angiopoietin-like 3 (ANGPTL3), a circulating inhibitor of lipoprotein lipase and endothelial lipase. ANGPTL3 inhibition leads to the increased activity of these enzymes, which reduces apoB-containing lipoproteins by promoting VLDL and remnant clearance [85]. The Efficacy and Safety of Evinacumab in Patients With Homozygous Familial Hypercholesterolemia (ELIPSE HoFH) phase 3 trial compared evinacumab to placebo in patients with HoFH not achieving LDL-C goal despite multiple LLT (77% on statins, 75% ezetimibe, 77% PCSK9 inhibitors, 25% lomitapide, and 34% lipid apheresis) and found a 49% placebo-controlled LDL-C reduction at 24 weeks [86]. The rate of adverse events was similar between the groups. In addition, the efficacy was unaffected by mutation type, with 72% placebo-controlled LDL-C reductions in those with virtually absent LDLR activity (n = 10). Evinacumab has FDA approval for use as an adjunct to other LLTs in adult and pediatric patients with HoFH aged 12 years and older.

## 7. Other Therapies

### 7.1 Lipoprotein Apheresis

Lipoprotein apheresis is the physical removal of apoB-containing lipoproteins from circulation. It lowers LDL-C by 50 to 76% acutely and is performed weekly or biweekly according to the severity of hypercholesterolemia [87]. No study has demonstrated clear benefits in survival or angiographic outcomes with lipoprotein apheresis [88, 89], although some studies indicate acute improvements in coronary microvascular dysfunction [90]. Despite its FDA approval, inadequate access, the burden of therapy, known side effects, and the growth of novel medical LLTs have led to heterogeneity in expert recommendations for apheresis initiation. The National Lipid Associated Expert Panel on FH recommendations, published in 2011, indicates lipoprotein apheresis in the following patients with inadequate response to maximal LLT after six months: HoFH with LDL-C ≥300 mg/dL; HeFH with LDL-C ≥300 mg/dL and 0–1 risk factors; HeFH with LDL-C ≥200 mg/dL and ≥2 risk factors or Lp(a) ≥50 mg/dL; and HeFH with LDL-C ≥160 mg/dL and ≥2 very high-risk characteristics (established CAD, other CV diseases, or diabetes) [91].

### 7.2 Surgical Interventions

Surgical options have been considered in patients with HoFH, particularly children, who fail to reach the LDL-C goal with maximally tolerated LLT and cannot receive regular lipoprotein apheresis. Liver transplantation has been used in patients with HoFH to provide functional hepatic LDLRs, therefore decreasing LDL-C and improving the efficacy of LLT. Early reports of pediatric patients with HoFH undergoing liver transplantation have shown impressive LDL-C reduction [92]. Similar limited data has shown LDL-C reductions after portacaval shunt [93] and partial ileal bypass [94], but their effect has been variable. Given the lack of long-term efficacy data, the higher risk of complications and side effects, and the rise in novel medical therapies, these options are infrequently utilized.

## 8. Conclusions

In summary, FH is the most common monogenic disorder in humans that results in elevated LDL-C levels from birth, leading to early-onset ASCVD. Although lipid-lowering therapies are effective in treating FH, the timely initiation of aggressive LDL-C-lowering treatment is crucial to reducing the morbidity and mortality associated with ASCVD. Regrettably, FH often goes undetected until after a cardiac event, and many individuals remain undiagnosed and undertreated. Therefore, raising awareness of FH among healthcare providers, patients, and the general public is critical to reducing morbidity and mortality associated with this condition.

## Abbreviations

AHA, American Heart Association; ANGPTL3, angiopoietin-like 3; apoB, apolipoprotein B; ASCVD, atherosclerotic cardiovascular disease; CAD, coronary artery disease; CASCADE, Cascade Screening for Awareness and Detection; CV, cardiovascular; CVD, cardiovascular disease; FDA, Food and Drug Administration; FH, familial hypercholesterolemia; HeFH, heterozygous familial hypercholesterolemia; HMG-CoA, hydroxymethylglutaryl CoA; HoFH, homozygous familial hypercholesterolemia; LDL, low-density lipoprotein; LDL-C, LDL cholesterol; LDLR, LDL receptor; LLT, lipid-lowering therapy; Lp(a), lipoprotein (a); MEDPED, Make Early Diagnosis to Prevent Early Death; PCSK9, proprotein convertase subtilisin/kexin type 9; VLDL-C, very low-density lipoprotein cholesterol.
